# Nutritional quality of Oyster Mushroom (*Pleurotus Ostreatus*) as affected by osmotic pretreatments and drying methods

**DOI:** 10.1002/fsn3.484

**Published:** 2017-06-20

**Authors:** Kumela D. Tolera, Solomon Abera

**Affiliations:** ^1^ Department of Post Harvest Management Jimma University Jimma Ethiopia; ^2^ Department of Food Science and Post Harvest Technology Haramaya University Dire Dawa Ethiopia

**Keywords:** Drying, mushroom, nutritional quality, osmotic pretreatments, preservation

## Abstract

This study was conducted to evaluate the effects of different levels of osmotic pretreatments prior to drying and different drying methods on nutritional quality of dried mushroom slices. The experiment consisted of sun, solar, and oven drying after dipping the slices in salt solutions of 5 and 10% concentrations for 50 minutes, the control being untreated mushroom sample. Significant differences in proximate composition were observed between the fresh and dried mushroom samples. The average mean value of crude protein, crude fat, crude fiber, ash, and carbohydrates of the fresh mushroom samples were 28.85, 2.47, 12.87, 9.76 and 48.16% as compared to 25.91, 2.18, 10.41, 10.91 and 42.14% for dried samples. Oven drying resulted in higher content of ash (11.06%) and carbohydrates (43.64%) and lower contents of crude protein (24.99%), crude fat (2.12%), and crude fiber (10.21%). The osmotic pretreatments significantly affected the composition of the dried mushroom samples. As salt concentration increased from 0 to 5 and 10%, the protein content reduced from 26.78 to 25.99 and 24. 95%, the fat reduced from 2.42 to 2.19 and 1.94, and fiber from 12.82 to 9.41 and 9.01%, respectively. Contrarily, the ash increased from 9.75 to 12.20%, and the carbohydrate from 38.16 to 43.08 and 45.18%, respectively.

## 1 INTRODUCTION

Mushrooms are edible fungi of commercial importance and their cultivation has emerged as a promising agro‐based land‐independent enterprise (Shivhare, Arora, Ahmed, & Raghavan, 2004). Obodai, Cleland‐Okine, and Vowotor (2003) reported that the cultivation of saprophytic edible mushrooms might be the only currently economical biotechnology for lignocelluloses organic waste recycling that combines the production of protein‐rich food with the reduction in environmental pollution. Mushrooms are increasingly being utilized as important food products for their significant role in human health, nutrition, and disease control (Chang & Miles, 1989).

Several species of mushrooms are of great importance because of their medicinal importance; for example, they are active against hypercholesterolemic conditions, hypertension, diabetes, cancer, infections etc. (Bobek & Ginter, 1993; Chang, 1996; Jose & Janardhanan, 2001; Manpreet, Soni, & Khanna, 2004; Nuhu Alam, Hossain, Khair, Amin, & Khan, 2007; Wang, Ooi, Ng, Chiu, & Chang, 1996; Yoshioka, Tabeta, Saitô, Uehara, & Fukuoka, 1985). Antitumor, antiviral, antithrombotic, and immunomodulating effects were also shown by mushrooms (Mau, Lin, & Chen, 2002). Mushrooms have antioxidant property due to presence of compounds like ergothioneine (Weigand‐Heller, Kris‐Etherton, & Beelman, 2012).

Mushrooms have been attracting attention of mankind since ancient times and use of mushroom as food is as old as human civilization. They have considerable importance in the human diet as they are rich in protein, nonstarchy carbohydrates, dietary fiber, minerals, and vitamin‐B and have no cholesterol, and negligible amount of fat. Mushroom proteins are of high quality and they contain an abundance of essential amino acids (Sadler, 2003).

The use of mushrooms may contribute significantly to overcome protein deficiency in the developing countries where good quality proteins from animal sources are either unavailable or unacceptable because of religious beliefs (Dunkwal, Jood, & Singh, 2007; Singh, Kumar, & Singh, 1995). Mushrooms can be a good supplement to cereals (Chang & Buswell, 1996) in enriching one's diet. Owing to their good nutritional and high digestibility values mushrooms are gaining importance in today's healthy diet.

Pleurotus as health promoter and environmental restorer is gaining more importance as compared to other medicinal mushrooms resulting in an upsurge in their research and development activities during the past two decades (Patel, Naraian, & Singh, 2012). The chemical nature of the bioactive compounds present in this mushroom includes: polysaccharides, lipopolysaccharides, proteins, peptides, glycoproteins, nucleosides, triterpenoids, lectins, lipids, and their derivatives.

In Ethiopia, mushroom cultivation is a very recent activity. In the past, mushroom consumption was confined to rural inhabitants who collected wild varieties from farmlands, forests, and around waste dumpsites. Dawit (2008) reported that for the first time small‐scale mushroom farms were started in 1997 by cultivation of oyster (*Pleurotus ostreatus*) mushroom. Later, the button (*Agaricus bisporus*) followed by the shiitake *(Lentinula edodes*) mushroom were introduced to the local market, specifically Addis Ababa. The local demand for mushrooms is steadily growing at about 36 tons per year (button 50%, oyster 40%, and shiitake 10%) at present.

According to Michael, Bultosa, and Pant (2010), the mushroom farms in Ethiopia still produce less than the local market demand. Production is increasing by about 20% annually. All mushrooms are sold as fresh mushrooms to supermarkets, restaurants, and international hotels in Addis Ababa. The supply of mushrooms to these hotels and restaurants is not consistent due to their poor farm management, low‐quality mushrooms, low productivity, and improper preservation techniques. As a result, they rely on imported canned mushrooms for the preparation of different dishes.

The mushrooms of the Pleurotus genus are delicate and sensitive, and start deteriorating within 1 day after the harvest (Apati, Furlan, & Laurindo, 2010). Mushrooms’ shelf life is limited to a few days under normal refrigeration conditions, which is a constraint on the distribution and marketing of fresh product, making extension of mushroom's shelf life a constant quest (Akbarirad, Kazemeini, & Shariaty, 2013). They have no cuticle to protect them from physical or microbial attack or water loss (Martine, Gaëlle, & Ronan, 2000). Various physiological and morphological changes occur after harvesting which makes the mushrooms unacceptable for consumption. Enzymatic activity in the mushrooms could lead to flavor deterioration (Mau et al., 1993). Browning, veil opening, weight loss, and microbial spoilage are the most common postharvest changes in mushrooms which often result in enormous economic losses.

Hence, development of appropriate postharvest technology like drying is crucial in order to extend their shelf life. Drying is a classical method of food preservation, based on the principle that the water activity of the product must be lowered until defined levels that guarantee the microbiological and physicochemical stability (Cao, Nishiyama, & Koide, 2003; Krokida, Karathanos, Maroulis, & Marinos‐Kouris, 2003; Lewicki & Jakubczyk, 2004). Drying is a relatively simple process that has been used for many years as a means to prolong the shelf life of food products. Mushrooms can be dried by different methods and dried mushrooms are convenient for long‐term storage and transportation.

Freeze‐drying produces a high‐quality product, but being an expensive process, its application for mushroom drying is limited (Giri & Prasad, 2013). Walde, Velu, Jyothirmayi, and Math (2006) reported that fluidized bed drying seems to be a promising method for drying mushrooms, when comparing the lower drying time and good‐quality products to the faster microwave drying. Although freeze‐drying produces a high‐ quality product, it is an expensive process and its application for mushroom drying is limited (Giri & Prasad, 2013). Kurozawa, Azoubel, Murr, and Park (2012) reported that the air drying of fresh and osmotically dehydrated mushroom in Brazil showed the influence of temperature and air velocity on drying kinetics and color of dried samples. Moreover, Aishah and Wan Rosli (2013) reported that low heat air blow drying method is recommended in reducing water activity and increasing proximate contents. Preserving mushroom in dried form can reduce the postharvest loss and extends their shelf life and oyster mushroom powders can be incorporated in various recipes for improving the nutritional status of vulnerable population in developing countries because of their high nutrient content (Muyanja, Kyambadde, & Namugumya, 2014).

In order to obtain the longest shelf life for a product, osmotic pretreatment is typically employed in association with other methods of food preservation including freezing, vacuum dehydration, and oven or freeze drying (Torringa, Esveld, Scheewe, van den Berg, & Bartels, 2001). Osmotic treatment is a method that is often used to maintain the quality and stability of dry food products (Krokida, Karathanos, & Maroulis, 2000). It helps to minimize the thermal damage on color, flavor, and texture, and prevents enzymatic browning of dried products (Islam & Flink, 1982). It is also used to facilitate the removal of water from the product and hence speedup the rate of drying and to reduce the dehydration time, which in turn minimizes the energy required for dehydration (Raoult‐Wack, 1994). Therefore, dehydration preceded by osmotic pretreatments appears to be a cost‐effective method of preservation (Rama & John, 2000).

Availability of mushrooms in its dried and safe form in the market could raise awareness in the public about the commercial and nutritional significance of this commodity and encourage the expansion of its production and consumption in the country. However, necessary information on drying of mushrooms under Ethiopian conditions and affordable technology in the country is lacking. This work was an extension of the efforts being made to promote and facilitate the commercial production and consumption of mushroom under the prevailing conditions through generating information on mushroom drying. The basic objective of this study was to determine the effect of osmotic pretreatments and drying methods on the nutritional quality of dried mushrooms.

## 2 MATERIAL AND METHODS

### 2.1 Sample preparation

Fresh oyster mushroom (*P.ostreatus*) (grown on cottonseed waste) was purchased from Sweet Mushroom Production and Supply Enterprise located in Addis Ababa. Sample preparation and drying experiments were done at Addis Ababa University, faculty of science and institute of technology. The rest of the works were done in food science and postharvest technology, animal nutrition, and soil science laboratories of Haramaya University, Ethiopia.

Mushrooms were harvested in the morning and sample preparation was started immediately after harvest. Fresh mushroom samples, free from blemishes, were washed thoroughly under running tap water to remove adhering soil particles. The cleaned mushroom samples were sliced manually into about 5 ± 1 mm thickness (Argyropoulos, Heindl, & Muller, 2008) using stainless steel knife prior to osmotic pretreatments.

### 2.2 Experimental design

The experiment was set in a 3 × 3 factorial arrangement involving three levels of osmotic pretreatments, that is, 0% (untreated), 5%, and 10% salt solution prior to drying and three drying methods (sun, solar, and oven drying), arranged in a completely randomized design (CRD) with three replications.

### 2.3 Osmotic pretreatments

Table salt (sodium chloride) was used as osmotic agent. The salt solutions were prepared at concentrations of 5 and 10% by dissolving required amounts of salt in distilled water. The prepared mushroom samples were soaked in salt solutions (Torringa et al., 2001) at ambient temperature for 50 minutes ensuring full coverage of the slices. The ratio of mushroom slices and the osmotic solution was 1:5 (w/v). After the osmotic pretreatments, the samples were withdrawn from the solution and dabbed gently with absorbent paper in order to remove excess water from the surface.

### 2.4 Drying experiments

After the osmotic pretreatments, the mushroom pieces were divided into three portions. The first portion was spread on aluminum foil sheets and dried under sun radiation by covering with very thin gauze to prevent the slices from dust and other foreign materials. The samples were stirred routinely to ensure uniform drying. It took 4–5 days to reach the required moisture content of about 12% depending on the ambient air condition during drying. The second portion of the prepared slices of mushroom samples was dried in a solar drier. The samples were spread on wire meshes and placed in the drying chamber. It took 3–4 days to reach the required moisture content of about 12%. The third portion of the prepared slices of mushroom were spread on stainless steel trays and placed in hot air oven at 60°C (Argyropoulos et al., 2008). The samples were dried until the required moisture content of about 12% was achieved. A fresh mushroom slice without osmotic pretreatments was also dried as control sample for each drying method to the same moisture content. The dried mushroom slices were packed and sealed in polyethylene plastic bags and stored in dry place at room temperature until they were required for analyses.

### 2.5 Data collection

In proximate composition determination, flour samples from both fresh and dried mushrooms were analyzed for chemical compositions. Fat, ash, and carbohydrates were analyzed using the procedures of AOAC (1995), while crude protein was analyzed using the procedures of AACC (2000).

### 2.6 Data analysis

The data were analyzed using SAS statistical package (version 9.1). Analysis of variance (ANOVA) was used to determine the significant differences in nutritional composition among the samples that received the various treatments. Fisher's least significant difference (LSD) test was used to compare the means’ significant differences.

## 3 RESULTS AND DISCUSSION

### 3.1 Proximate composition of fresh mushroom

The data of the proximate composition of fresh oyster mushroom (*P. ostreatus)* grown on cottonseed waste as a substrate is presented in Table 1. The moisture content was 88.75%, which is in the range reported by Manzi, Gambelli, Marconi, Vivanti, and Pizzoferrato (1999) for the fruiting bodies of fresh *P. ostreatus* cultivated on different lignocellulosic agro‐wastes. Similar results were also reported by Hassan and Medany (2014) and Patil, Ahmed, Telang, and Baig (2010). The results of crude protein and crude fat obtained from the fresh samples were 28.85 and 2.47%, respectively, which are in the range reported by Rashad, Abdou, Mahmoud, and Nooman(2009). Hassan and Medany (2014) also reported 28.52% of crude protein for fresh *P. ostreatus*. On the other hand, for the dried samples, corresponding mean values were 25.91 and 2.18%, respectively, which are significantly (*p *<* *.05) lower than those of the fresh samples. Similarly, crude fiber content of 12.87% and 10.41% was recorded for the fresh mushrooms and dried samples, respectively. The results of this study is close to the values reported by Michael, Bultosa, and Pant (2011). Total ash content of dried samples were significantly (*p *<* *.05) lower than that in the fresh sample with average mean values of 9.76 and 10.91% for fresh and dried samples, respectively.

**Table 1:** Proximate composition of fresh and dried mushroom slices

Mushroom samples	Moisture content (%)	Crude protein (% db)	Crude fat (% db)	Crude fiber (% db)	Total ash (% db)	Carbohydrate (%)
Fresh	88.75 ± 0.02	28.85 ± 0.04	2.47 ± 0.01	12.87 ± 0.02	9.76 ± 0.02	48.16 ± 0.03
Dried	8.45 ± 1.65	25.91 ± 1.28	2.18 ± 0.21	10.41 ± 1.84	10.91 ± 1.22	42.14 ± 3.45

### 3.2 Effect of drying methods and osmotic concentrations on proximate composition of dried mushroom slices

#### 3.2.1 Moisture content

Moisture content data of dried mushroom slices are presented in Table 2. The results of samples dried by different drying methods were significantly (*p *<* *.05) different from each other. The higher moisture content (9.58%) was recorded for in the sun‐dried sample, while the lower value, (7.77%) was obtained for solar dried sample. This might be due to moisture absorption of the dried samples from the environment. In sun drying methods, case hardening might occur and causing the moisture content to be higher than samples dried by other drying methods under similar treatment conditions. Similar results were also reported by Hassan and Medany (2014), who reported 7.93% of moisture content of *P. ostreatus* pretreated in NaCl prior to drying.

**Table 2:** Main effect of drying methods and osmotic pretreatments on proximate composition of dried mushroom

Variable	Moisture content (%)	Crude protein (% db)	Crude fat (% db)	Total ash (% db)	Crude fiber (% db)	Carbohydrate (% db)
Drying Methods						
Ov	7.99 ± 1.19^b^	24.99 ± 1.14^c^	2.12 ± 0.18^b^	11.06 ± 1.88^a^	10.21 ± 2.08^b^	43.64 ± 2.52^a^
Sn	9.58 ± 1.74^a^	27.14 ± 0.41^a^	2.22 ± 0.23^a^	10.93 ± 0.36^b^	10.14 ± 1.47^b^	39.99 ± 3.04^c^
Sr	7.77 ± 1.48^c^	25.59 ± 1.04^b^	2.21 ± 0.22^a^	10.74 ± 1.06^c^	10.90 ± 2.01^a^	42.79 ± 3.85^b^
Osmotic conc.						
0%	10.07 ± 1.09^a^	26.78 ± 0.56^a^	2.42 ± 0.06^a^	9.75 ± 0.70^c^	12.82 ± 0.64^a^	38.16 ± 1.94^c^
5%	8.57 ± 0.88^b^	25.99 ± 1.11^b^	2.19 ± 0.10^b^	10.77 ± 0.45^b^	9.41 ± 0.68^b^	43.08 ± 1.78^b^
10%	6.70 ± 0.70^c^	24.95 ± 1.37^c^	1.94 ± 0.02^c^	12.20 ± 0.87^a^	9.01 ± 0.47^c^	45.18 ± 1.60^a^
Mean	8.45 ± 1.65	25.91 ± 1.28	2.18 ± 0.21	10.91 ± 1.22	10.41 ± 1.84	42.14 ± 3.45
CV	2.20	0.55	1.19	1.11	1.63	0.67

Values are mean ± SD and mean values followed by the same letters in a column are not significantly different at (*p* < .05); U, Untreated sample; Ov, Oven drying; Sn, Sun drying; Sr, Solar drying; CV, coefficient of variance.

Significant (*p *<* *.05) differences in moisture content were observed for the mushroom slices pretreated with two levels of salt concentration and untreated sample prior to drying. The highest value (10.07%) was recorded for untreated mushroom slices, whereas the lowest moisture content (6.70%) was recorded for the sample pretreated in 10% sodium chloride solution. This might be due to osmotic pressure occurring in osmotic solution which pushed out more moisture from the interior tissues even before drying. A similar result was also reported by Tulek (2011), who reported 10% moisture content for dried *P.ostreatus*.

#### 3.2.2 Crude protein content

Crude protein content data of dried mushroom slices are presented in Table 2. Values of samples dried by different drying methods were significantly (*p *<* *.05) different from each other. Significantly higher protein content (27.14%) was observed for the sample dried in the sun when compared to those of the other treatments. Lower protein content, 24.99%, was observed in the oven‐dried product. It was found that though a medium temperature of 60°C was used in the oven drying method, it affected the protein content significantly than the other drying methods. Similarly, Arumuganathan, Manikantan, Indurani, Rai, and Kamal (2010) reported that temperature in the order of 60°C could result in denaturation of protein leading to a reduction in protein content of oyster mushroom. However, Yang, Lin, and Mau (2001) reported lower protein content (23.9%) as compared to this study. This variation might be due to the difference in mushroom‐growing substrates. The lower protein content of dried oyster mushroom may be due to leaching out during steeping and/or loss throughout browning reactions. In general, drying process causes a considerable decrement in protein content (Hassan & Medany, 2014).

Concerning pretreatments done to mushroom slices prior to drying, statistically significant (*p *<* *.05) differences in the crude protein content were observed among the samples. The highest value of crude protein (26.78%) was recorded for the untreated dry mushroom samples followed by the (25.99%) for those treated with 5% salt concentration. The lowest crude protein content, 24.95%, was obtained from the mushroom slices that were soaked in 10% salt solution. This might be due to the much greater protein solubilization during osmotic treatment (brining) at higher concentration of salt which resulted in the removal of more water from the tissues (Muyanja et al., 2014). The crude protein results of this study is close to the finding of Hassan and Medany (2014), who reported 26.83% of crude protein for *P. ostreatus* pretreated in NaCl and dried at 50°C.

Statistically, no significant difference in crude protein content was observed between the untreated samples and those treated with 5% salt solution and subjected to sun drying methods (Table 2), with the mean values of 27.32 and 27.46%, respectively. These were statistically the highest values. The lowest value of crude protein was observed for samples treated at 10% osmotic solution combined with oven drying.

#### 3.2.3 Crude fat content

Crude fat content data of the dried mushroom are presented in Table 2. Sun‐dried and solar‐dried samples exhibited 2.22% and 2.21% of crude fat content with no significant difference between them but were statistically higher than that of the oven‐dried samples (2.12%). The results obtained in this study were close to that obtained by (Reguła & Siwulski, 2007), who reported 2.66% crude fat for dried oyster mushroom.

Table 2 also shows the values of crude fat as affected by the concentration of the osmotic solutions. Statistically significant (*p *<* *.05) differences were noted among the values belonging to three levels of salt solutions. The control sample had 2.42% and those of the samples treated with 5% and 10% salt concentration were 2.19 and 1.94%, respectively.

The interaction effect of drying methods and osmotic concentrations on crude fat content of dried oyster mushroom (*P. ostreatus*) was significant (*p *<* *.05) (Table 3). The highest three fat contents were 2.48, 2.43, and 2.35% of the control (0% salt) samples dried in the sun, in solar dryer, and in oven, respectively, with significant differences among three of them. Contrarily, the lowest crude fat contents (1.93, 1.95, and 1.96%; with no statistical difference) were observed in the samples subjected to the 10% salt solution and dried by the three drying methods. The data also showed that as salt concentration increased, the crude fat content decreased. Osmotic treatment had strong influence on the interactions with drying methods.

**Table 3:** Interaction effect of drying methods and osmotic pretreatments on proximate composition of dried mushroom

DT	OT	Moisture content (%)	Crude protein (% db)	Crude fat (% db)	Total ash (% db)	Crude fiber (% db)	Carbohydrate (% db)
Ov	0%	9.20 ± 0.00^c^	26.07 ± 0.05^d^	2.35 ± 0.02^c^	8.89 ± 0.12^g^	12.95 ± 0.27^b^	40.53 ± 0.36^d^
Ov	5%	8.26 ± 0.07^d^	25.37 ± 0.09^e^	2.06 ± 0.04^e^	11.06 ± 0.02^c^	9.15 ± 0.08^f^	44.09 ± 0.14^b^
Ov	10%	6.49 ± 0.09^f^	23.53 ± 0.22^g^	1.95 ± 0.00^f^	13.22 ± 0.31^a^	8.52 ± 0.09^h^	46.29 ± 0.16^a^
Sn	0%	11.50 ± 0.15^a^	27.32 ± 0.19^a^	2.48 ± 0.05^a^	10.47 ± 0.12^d^	12.05 ± 0.08^c^	36.18 ± 0.53^f^
Sn	5%	9.69 ± 0.13^b^	27.46 ± 0.12^a^	2.23 ± 0.02^d^	11.07 ± 0.01^c^	8.81 ± 0.02^gh^	40.74 ± 0.26^d^
Sn	10%	7.54 ± 0.48^e^	26.64 ± 0.25^c^	1.96 ± 0.01^f^	11.24 ± 0.05^c^	9.56 ± 0.19^e^	43.06 ± 0.20^c^
Sr	0%	9.50 ± 0.15^cb^	26.94 ± 0.05^b^	2.43 ± 0.03^b^	9.91 ± 0.01^f^	13.47 ± 0.14^a^	37.77 ± 0.16^e^
Sr	5%	7.74 ± 0.05^e^	25.13 ± 0.03^e^	2.27 ± 0.02^d^	10.17 ± 0.04^e^	10.26 ± 0.33^d^	44.42 ± 0.33^b^
Sr	10%	6.08 ± 0.06 ^g^	24.68 ± 0.07^f^	1.93 ± 0.01^f^	12.15 ± 0.05^b^	8.96 ± 0.03g^f^	46.20 ± 0.03^a^
Mean		8.45 ± 1.65	25.91 ± 1.28	2.18 ± 0.21	10.91 ± 1.22	10.41 ± 1.84	42.14 ± 3.45
CV		2.20	0.55	1.19	1.11	1.63	0.67

CV, values are mean ± SD and mean values followed by the same letters in a column are not significantly different at (*p* < .05); U, Untreated sample; Ov, Oven drying; Sn, Sun drying; Sr, Solar drying; CV, coefficient of variance.

#### 3.2.4 Crude fiber content

Crude fiber content data of dried mushroom are presented in Table 2. Higher value (10.90%) of crude fiber content was obtained from the solar‐dried mushroom samples compared to those (10.21% and 10.14%) of samples dried in oven and the sun, respectively. According to Dunkwal et al. (2007), sun‐dried and oven‐dried *Pleurotus sajor‐caju* contained 12.59% and 12.58% crude fiber, respectively.

The mushroom slices pretreated with different levels of salt solution prior to drying were observed to have significantly (*p *<* *.05) different amount of crude fiber. The highest value (12.82%) was recorded for the control samples, whereas the lowest (9.01%) was recorded for the sample subjected to 10% salt solution prior to drying. Generally, the crude fiber content observed in this study decreased with increase in the concentration of salt solution.

The interaction of osmotic treatments and drying methods significantly (*p *<* *.05) affected the crude fiber content of mushroom slices (Table 3). The highest value (13.47%) was recorded for the untreated mushroom sample dried by solar drying techniques, whereas the lowest crude fiber content (8.52%) was recorded for those soaked in 10% salt concentration combined with oven drying method. This showed that concentration had strong influence on the interaction with the drying methods regarding the crude fiber content.

#### 3.2.5 Total ash content

The different drying methods significantly (*p *<* *.05) affected the total ash contents of dried mushroom slices (Table 2). The value for oven‐dried mushroom was 11.06% and is statistically higher than the 10.93 and 10.74% recorded for those dried in the open sun and solar drier, respectively.

The mushroom slices pretreated with different levels of salt concentration and those untreated prior to drying were observed to have significant (*p *<* *.05) differences in total ash content. The highest value (12.20%) was recorded for the mushroom slices that were immersed in 10% sodium chloride solution, whereas the lowest ash level (9.75%) was recorded for the untreated dry sample.

The interaction of drying methods with osmotic treatments significantly (*p *<* *.05) affected the ash content of mushroom slices (Table 3). The highest ash content (13.22%) was recorded for the sample soaked in 10% salt concentration and dried by oven. The lowest ash content (8.89%) was observed for the untreated sample, which was subjected to oven drying.

The ash content of the dried mushrooms increased with increase in the strength of the salt solution used for osmotic treatment from 0 to 10%. The increase in ash content is perhaps due to sodium in the solution that might have migrated into the mushroom slices when the water drained out. This is due to the simultaneous process of water and solute diffusion in osmotic dehydration (Krokida & Marinos‐Kouris, 2003; Lerici, Pinnavaia, Rosa, & Bartolucci, 1985).

#### 3.2.6 Carbohydrate content

Carbohydrate in untreated and pretreated samples of dried *P. ostreatus* was significantly (*p *<* *.05) affected by the method of drying (Table 2). The highest value (43.64%) of carbohydrate content was recorded for the oven‐dried mushroom samples, whereas the lowest value (39.99%) was recorded for the samples dried in open sun.

Similarly, the mushroom slices pretreated with different levels of salt concentrations were significantly (*p *<* *.05) different from one another in carbohydrate content. The highest value (45.18%) was recorded for the mushroom slices that were soaked in 10% sodium chloride solution, whereas the lowest amount (38.16%) was observed for the untreated sample prior to drying.

The interaction of the two factors revealed significant (*p *<* *.05) differences on the available carbohydrate content of dried mushroom slices (Table 3). The highest values of available carbohydrate (46.29% and 46.20%) were recorded for the mushroom samples soaked in 10% salt solution and dried in oven and solar dryers, respectively, while the lowest (36.18%) was observed for untreated sun‐dried mushroom sample. Mushroom samples subjected to 5% osmotic concentration combined with any of the drying methods resulted in intermediate values. Carbohydrate content of dried mushroom slices increased with the strength of osmotic concentration regardless of drying methods. This might be due to the increase in total ash content with the strength of salt concentration.

## 4 CONCLUSIONS

The different levels of osmotic pretreatments and drying methods resulted in statistically significant difference in nutritional quality of dry mushrooms. Both the solar drying methods and 5% osmotic concentration, (alone or in combination) resulted in the best proximate composition of dried mushroom slices. The solar drying methods and lowest concentration (5%) of osmotic solution are advantageous in terms of product quality when compared to oven drying methods and 10% salt solution. In addition, unlike sun drying, the mushrooms being dried in the solar and oven drier were completely protected from rain, insects, and dust, and the dried mushrooms were of high‐quality products.

In conclusion, the application of appropriate and sound postharvest practices of storage and processing like drying with lower concentration of osmotic agent are needed to solve the perishability problem of mushroom and hence to increase the potential of mushroom cultivation and commercialization in countries like Ethiopia where mushroom cultivation is very low. As a future line of work, intensive research and technology transfer of optimizing pretreatments prior to drying and drying technologies in mushroom processing and preservation must be encouraged. Furthermore, researches need to evaluate other different species of edible mushrooms for optimizing processing and preservation methods for the distribution of quality dried mushrooms so as to popularize mushroom production, preservation, and consumption in countries like Ethiopia where mushroom production and consumption habit is very low. Researches also need to investigate the effect of different types of packaging materials and storage times on the physicochemical quality of dried mushroom for its long‐term storage.

## ACKNOWLEDGMENTS

Firstly, the authors are thankful to Ministry of Education, Federal Democratic Republic of Ethiopia for granting financial support of the experiments and the Department Food Science and Postharvest Technology of Haramaya University for their support in allowing the use of their laboratory facilities. The authors also thank the Department of Mechanical Engineering, and Chemistry, Addis Ababa University, Ethiopia for their support in allowing the use of their laboratories and drying facilities. The authors are also thankful to the Sweet Mushroom Production and Supply Enterprise located in Addis Ababa, Ethiopia for providing the mushrooms.

## CONFLICT OF INTEREST

None declared.
